# A meta-analysis of total thyroidectomy and lobectomy outcomes in papillary thyroid microcarcinoma

**DOI:** 10.1097/MD.0000000000036647

**Published:** 2023-12-15

**Authors:** Jinzhe Bi, Hao Zhang

**Affiliations:** a Department of General Surgery, The First Affiliated Hospital of Hainan Medical University, Haikou, China.

**Keywords:** lobectomy, meta-analysis, papillary thyroid microcarcinoma, thyroidectomy

## Abstract

**Introduction:**

Current research on the most effective surgical method for papillary thyroid microcarcinoma is in dispute. Specifically, whether a total thyroidectomy (TT) is superior to a thyroid lobectomy (LT) in terms of recurrence rate, postoperative complications, and recurrence-free survival is an issue to be addressed. The objective of this study was to compare TT with LT in terms of recurrence, postoperative complications, and recurrence-free survival.

**Methods:**

In accordance with the Preferred Reporting Items for Systemic Reviews and Meta-Analyses standards, the PubMed, Embase, web of science and the Cochrane Library database were searched for relevant studies comparing TT versus LT. By pooling the relative risks (RR) of the 2 surgical procedures, perioperative results of the 2 group can be estimated. Recurrence-free survival was calculated from hazard ratios between the 2 surgical group.

**Results:**

This meta-analysis included 8 studies involving 16,208 patients. In the TT group, there were fewer recurrences than in the LT group. (RR = 0.68; 95% confidence interval [CI], 0.39 to 1.18; *P* = .001). In subgroup analyses based on country and sample size, there were no significant differences between the 2 groups for the recurrence rates. We found that patients that underwent LT had lower total complication rates (RR = 15.12; 95% CI, 8.89 to 25.73; *P* = .009), wound recurrent laryngeal nerve injury and hypocalcemia. In terms of survival, TT can provide better recurrence-free survival than LT, with a hazard ratios of 0.57 (95% CI 0.36 to 0.90; *P* = .003).

**Conclusion:**

Comparing TT with LT, no statistical difference was found in recurrence rates between the 2 groups. In addition, the analysis showed a slight improvement in long-term recurrence-free survival for patients who underwent TT than for those who underwent LT, a finding with potential clinical implications for management decisions on papillary thyroid microcarcinoma treatment.

## 1. Introduction

In recent years, the incidence of thyroid cancer has been on the rise globally, with papillary thyroid microcarcinoma (PTMC) accounting for more than 50% of all newly diagnosed thyroid cancers.^[1,2]^ According to the World Health Organization definition, PTMC refers to papillary thyroid carcinoma whose tumor diameter is <10 mm.^[3]^ Currently, the primary treatment for papillary thyroid carcinoma is surgical intervention, which varies based on the individual condition, including either lobectomy (LT) or total thyroidectomy (TT).^[4]^ The disease-specific mortality rate after surgical resection is < 1%, so the vast majority of patients with PTMC have a good prognosis.^[5]^ However, there is still controversy regarding the extent of surgical intervention for PTMC. Whether PTMC has a good prognosis or whether it still runs the risk of recurrence and metastasis is a matter of some debate. According to some scholars, PTMC has a good prognosis, but still carries a risk of recurrence and metastasis.^[6–9]^ Therefore, these researchers advocate for a TT, which includes the right lobe, the left lobe, the pyramidal lobe, and the isthmic lobe. The central lymph nodes of the thyroid are also dissected, if necessary. In contrast, some other scholars argue that TT is overtreatment since there is insufficient evidence that it can reduce the death and recurrence risk in PTMC patients, and LT can achieve the therapeutic effect of TT.^[10–12]^ In the latest guidelines from the American Thyroid Association, using LT alone may be sufficient for the treatment of PTMC. However, the majority of its recommendations are based on studies of papillary thyroid cancer.^[4]^

The favorable prognosis of PTMC has prompted clinicians to develop a new understanding and reconsider the surgical approach for PTMC. In this meta-analysis, we compared the clinical outcomes of PTMC patients undergoing both TT and LT procedures, laying the foundation for the selection of the most effective surgical strategy.

## 2. Patients and methods

### 2.1. Search strategy and selection criteria

We searched PubMed, Embase, Web of Science, and the Cochrane Library databases. For the search strategy that led to the discovery of this article, we used the following keywords: *papillary thyroid microcarcinoma, PTMC, total thyroidectomy and lobectomy.* To find articles with information on these terms, the Boolean operators “and” and “or” were used. References in original articles and review were manually checked for additional studies. Only English articles are screened. For inclusion in this meta-analysis, we used the following criteria: Comparison of TT with LT in the treatment of PTMC, there is at least 1 outcome data required for this study, such as recurrence rate, complications (we defined complications as recurrent laryngeal nerve injury and hypocalcemia following surgery). Exclusion criteria included: The articles covered topics not related to the PTMC, duplicate literature, case report, reviews, comments, abstracts, no data were provided for this study, and animal experiment.

### 2.2. Study quality assessment

All included articles are evaluated and documented. Based on the Newcastle–Ottawa scale (NOS) evaluation, the included studies were evaluated for quality.^[13]^ NOS comprises the selection of study groups, the comparability between the groups, and assessment of outcomes. Each study is scored out of 9 points. Studies scoring 7 or more were considered high quality. Otherwise, studies were excluded from the final meta-analysis.

### 2.3. Data extraction

In this study, 2 reviewers extracted the data independently and checked their accuracy with other reviewers. After discussion, discrepancies were resolved by consensus. We extracted essential information such as first author, publication year, country, patients’ number in each group, type of study, tumor size, median follow-up time, recurrence rates and complications for the studies. Recurrent laryngeal nerve injuries and hypocalcemia were among the complications we extracted.

### 2.4. Statistical analyses

Analysis of the data was conducted using STATA 16.0 software. In meta-analyses, the relative risk (RR) should be used for dichotomous data, and the mean difference should be used for continuous data. According to Parmar et al,^[14]^ the hazard ratios (HR) and its variance were extracted and estimated. The 95% confidence interval (CI) is used with pooled estimates. Statistical significance was defined as *P* < .05. When *P* > .10 or *I*^2^ < 50%, a low inter-literature heterogeneity was indicated, and a fixed-effects model was used for the Meta-analysis. If *P* < .1 or *I*^2^ > 50%, indicating high heterogeneity between the literatures, then a random effects model was used. For risk factors with high heterogeneity, 1 sensitivity analysis was carried out by excluding literature methods for different types of included studies. We evaluated publication bias using the drawing funnel mapping method as well as Egger test.^[15]^

## 3. Results

### 3.1. Search results

**Figure 1.**
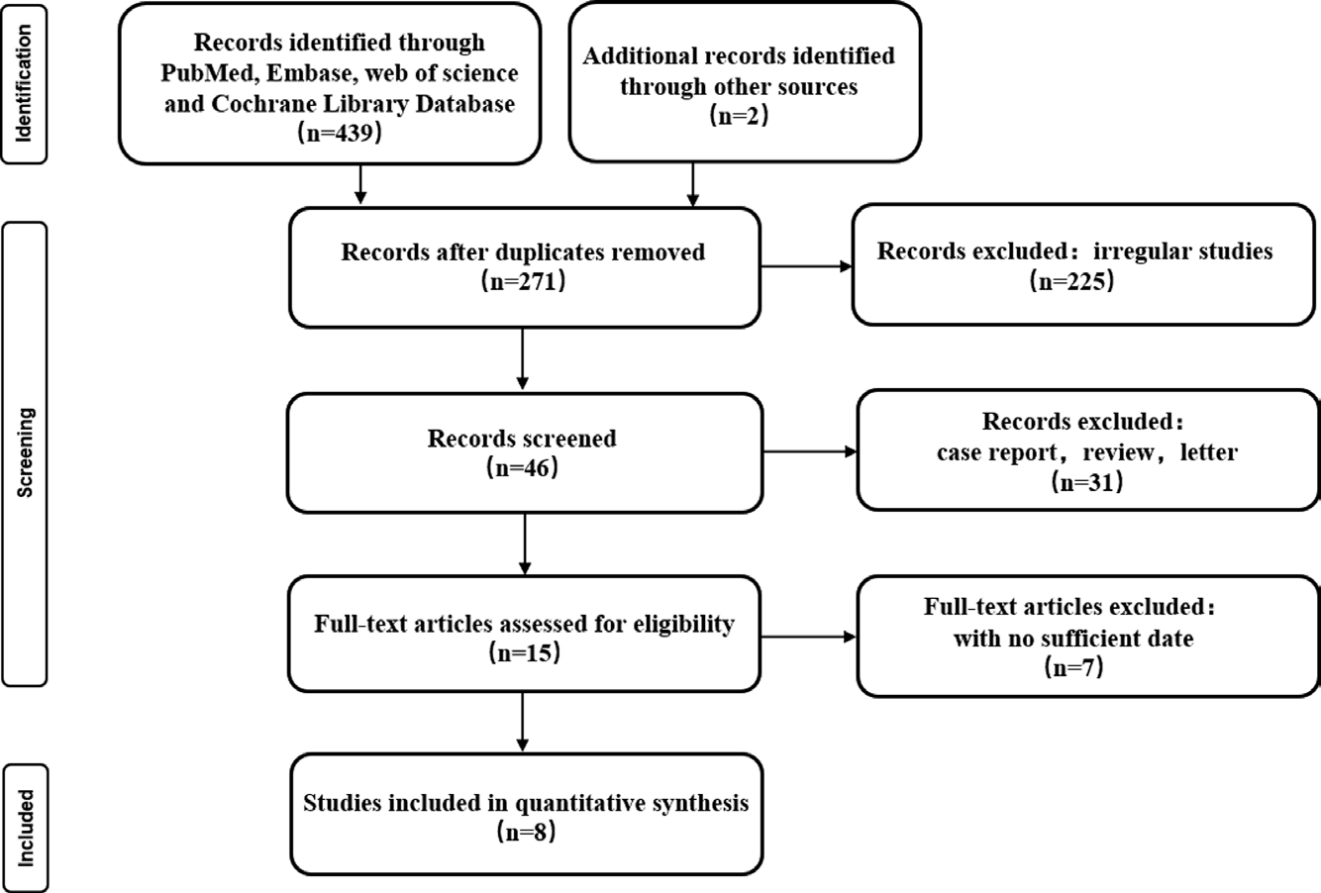
Study selection and literature search flow chart.

Initially, 441 articles were obtained through database searches and other methods. Following reading the titles and abstracts of 426 articles, we excluded them because they failed to meet our inclusion criteria. After carefully reviewing the full texts of 15 studies, 9 articles were excluded. Finally, this meta-analysis incorporated data from 8 studies (Fig. 1).^[16–23]^ A summary of the characteristics of all the studies is provided in Table 1. In this meta-analysis, we evaluated the quality of the included cohort studies by using NOS methodology (Table 2).

**Table 1:** Characteristics of the included studies.

Authors/yr of publication	Country	Female (%)	Age, yr (mean ± SD or range)		Study design	Number of patients		Mean follow-up time (yr)		Mean tumor diameter (cm)	
			TT	LT		TT	LT	TT	LT	TT	LT
Hay/2008	USA	627 (69.7)	46		Cohort	765	125	17.2		0.7	
Lee/2013	Korea	908 (89.7)	45.3		Cohort	506	506	11.8		0.61	0.59
Donatini/2016	France	717 (81.4)	48.5 (19–90)	44.2 (11–80)	Cohort	251	69	10.8	11.2	0.78	0.71
Dobrinja/2017	Italy	80 (76.2)	54 (12–77)	56 (30–79)	Cohort	86	19	4.8	5.3	0.53	0.6
Kim/2017	Korea	7057 (81.3)	48.3 ± 10.4	45.5 ± 10.5	Cohort	5387	3289	5.4		0.6 ± 0.2	0.5 ± 0.2
Kwon/2017	Korea	1256 (91.3)	47 (41–54)		Cohort	688	688	8.5		0.6	0.6
Xu/2018	China	2739 (75.9)	47.5 ± 12.0		Cohort	1868	1706	5.7		0.6 ± 0.3	
Jeon/2019	Korea	226 (88.6)	47.65 ± 10.39	47.65 ± 10.39	Cohort	128	127	8	7.9	0.67 ± 0.2	0.63 ± 0.2

LT = lobectomy, TT = total thyroidectomy.

**Table 2:** Meta-analysis of observational studies using Newcastle–Ottawa scale (NOS).

*Authors/yr of publication*	Newcastle–Ottawa scale								
	Representativeness of the exposed cohort	Selection of the unexposed cohort	Ascertainment of exposure	Outcome of interest not present at start of study	Control for important factor or additional factor	Outcome assessment	Follow-up long enough for outcomes to occur enough for outcomes to occur	Adequacy of follow-up of cohorts	Total scores
Hay/2008	☆	☆	☆	☆	☆	☆	☆	☆	8
Lee/2013	☆	☆	☆	☆	☆☆	☆	☆	☆	9
Donatini/2016	☆	☆	☆	☆	☆	☆	☆	☆	8
Dobrinja/2017	☆	☆	☆	☆	-	☆	☆	☆	7
Kim/2017	☆	☆	☆	☆	☆	☆	☆	☆	8
Kwon/2017	☆	☆	☆	☆	☆	☆	☆	☆	8
Xu/2018	☆	☆	☆	☆	☆	☆	☆	☆	8
Jeon/2019	☆	☆	☆	☆	☆	☆	☆	☆	9

### 3.2. Characteristics of the studies

Study publication dates for all 8 eligible studies range from 2008 to 2019. Studies were conducted in South Korea,^[17,19,21,22]^ France,^[18]^ Italy,^[20]^ China,^[23]^ and the United States.^[16]^ All studies were retrospective cohort studies. In all the included articles, the quality of the research was high. A total of 16,208 patients were enrolled in the 8 studies, of which 9679 patients underwent TT and 6529 patients underwent LT.

### 3.3. Analyses of recurrence rates pooled

**Figure 2.**
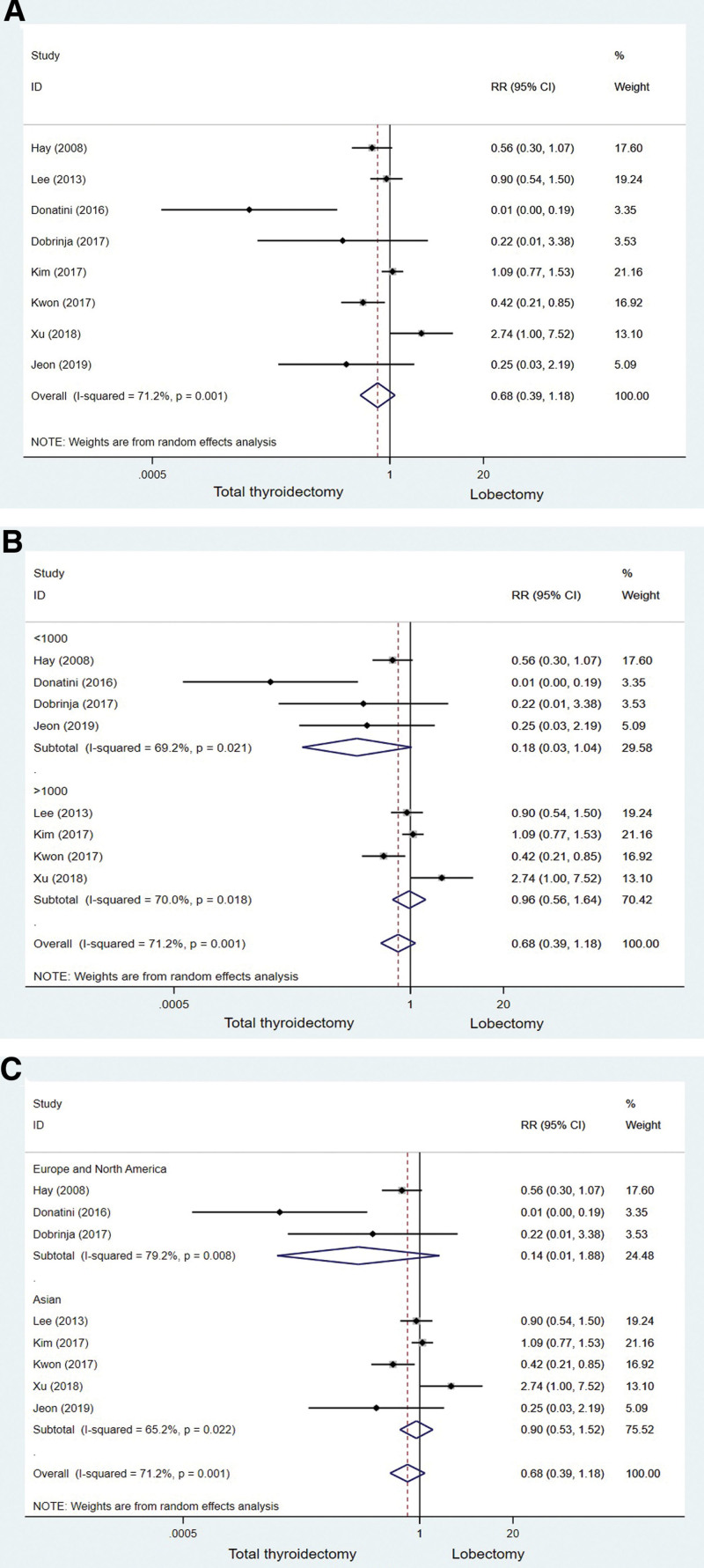
The forest plot of studies comparing total thyroidectomy and lobectomy for recurrence rates in PTMC patients. (A) Including all group of patients. (B) Subgroup analysis based on Sample size. (C) Subgroup analysis based on Country. PTMC = papillary thyroid microcarcinoma.

An original summary of basic characteristics such as the year of publication and sample size of the included literature is presented in Table 1. Comparatively to groups undergoing LT, TT groups saw low rates of tumor recurrence. In a random-effects model analysis, the pooled estimate RR for the difference in recurrence rate between the TT and LT groups was 0.68 [95% CI: 0.39–1.18, *P* = .001] (Fig. 2A). It was determined that there was no significant difference in the recurrence rate between groups in the pooled analysis above, however the *I*^2^ and *P* value were 71.2% and .001 respectively in the *Q* test. Evidence of significant heterogeneity among the studies. Subgroup analyses based on sample size found no significant differences between the 2 groups (sample size < 1000: RR = 0.18, 95% CI: 0.03–1.04, *P* = .021; sample size > 1000: RR = 0.96, 95% CI, 0.56 to 1.64, *P* = .018) (Fig. 2B). The 2 groups did not differ significantly from 1 another on the basis of country-based subgroup analysis. (Europe and North America: RR = 0.14, 95% CI: 0.01–1.88, *P* = .008; Asian: RR = 0.90, 95% CI: 0.53–1.52, *P* = .022) (Fig. 2C).

### 3.4. Complications totaled

**Figure 3.**
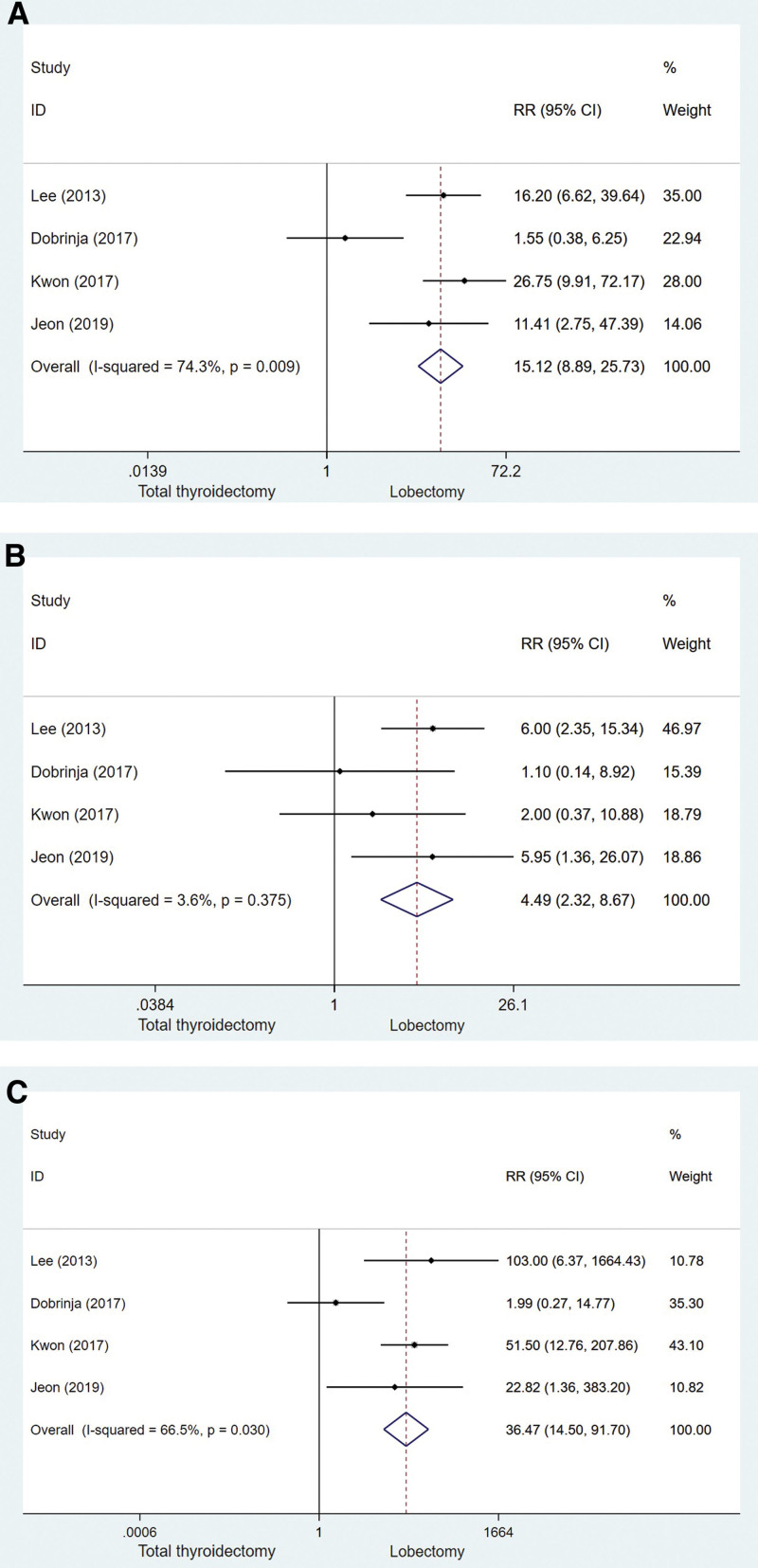
Complications of thyroidectomy versus lobectomy with PTMC (A) Including all group of patients. (B) Recurrent laryngeal nerve injury. (C) Hypocalcemia. PTMC = papillary thyroid microcarcinoma.

Our study included 4 studies to determine the total complication rate. We defined complications as recurrent laryngeal nerve injury and hypocalcemia following surgery. A comparison of the total complications of thyroidectomy and LT is shown in Figure 3A (RR = 15.12, 95% CI: 8.89–25.73, *P* = .009). According to the data, the LT group had lower rates of perioperative complications than the TT group. Recurrent laryngeal nerve injury and hypocalcemia were also analyzed for overall complications. Significant differences in recurrent nerve injury between the TT and LT group are shown in Figure 3B (RR = 4.49, 95% CI, 2.23–8.67, *P* = .375). Hypocalcemia also showed significant differences between the TT group and the LT group, as shown in Figure 3C (RR = 38.47, 95% CI, 14.50–91.70, *P* = .030). Overall, TT-treated patients experienced higher complications than TL-treated patients, whether it was recurrent laryngeal nerve injury or hypocalcemia.

### 3.5. Recurrence-free survival

**Figure 4.**
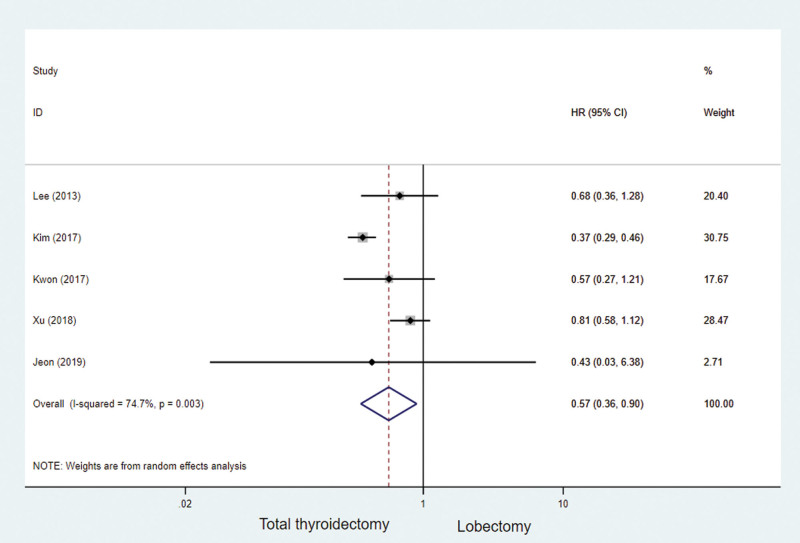
Survival outcomes for all included studies for the total thyroidectomy and lobectomy.

Based on the methods described by Parmar et al,^14^ we performed survival analyses on 5 studies and calculated HRs based on survival data. For the survival rate, expressed as a comparison between thyroidectomy and LT, the HRs was 0.57 (95% CI: 0.36–0.90; *P* = .003, Fig. 4), These results suggest that TT actually has better recurrence-free survival than LT.

### 3.6. Sensitivity analysis

**Figure 5.**
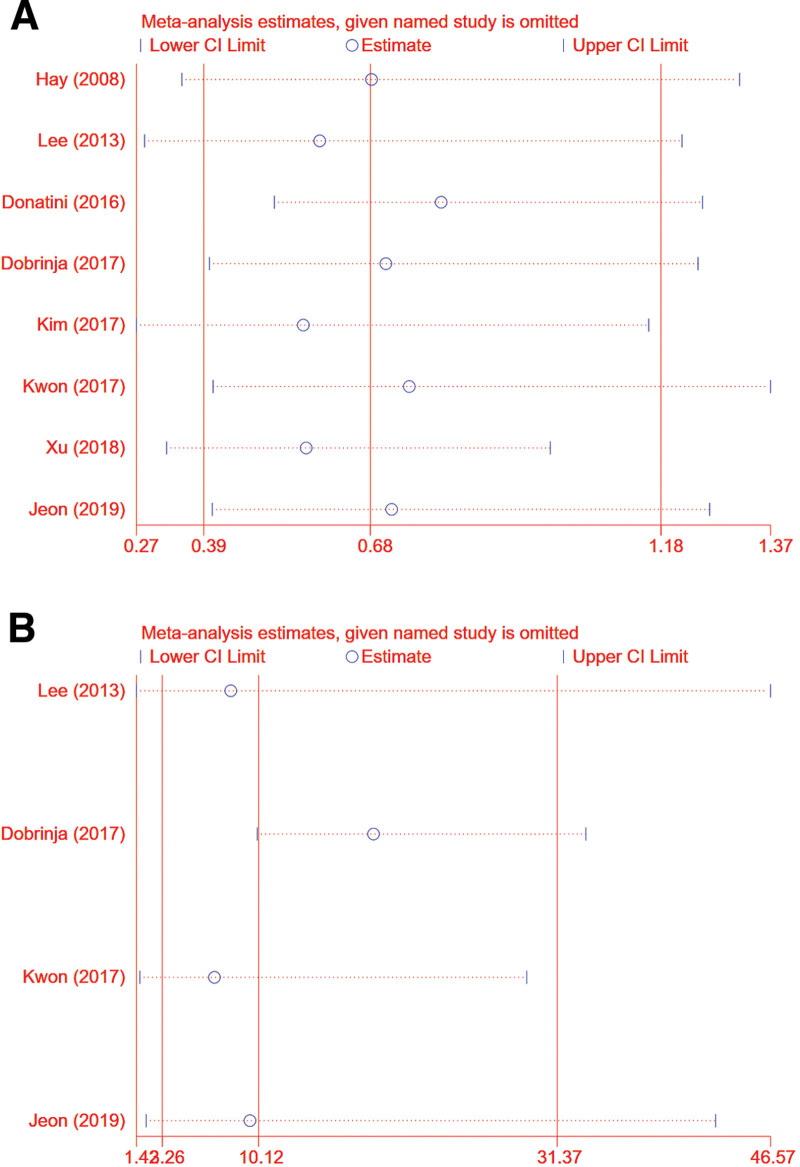
Sensitivity analysis. (A) Recurrence rates (B) total complication.

We performed sensitivity analyses in order to determine the impact of individual studies on the overall RR. Results showed that no individual study significantly influenced the overall RR (Fig. 5A and B shows recurrence and total complication rates, respectively). This suggests that our overall results are stable.

### 3.7. Publication bias

**Figure 6.**
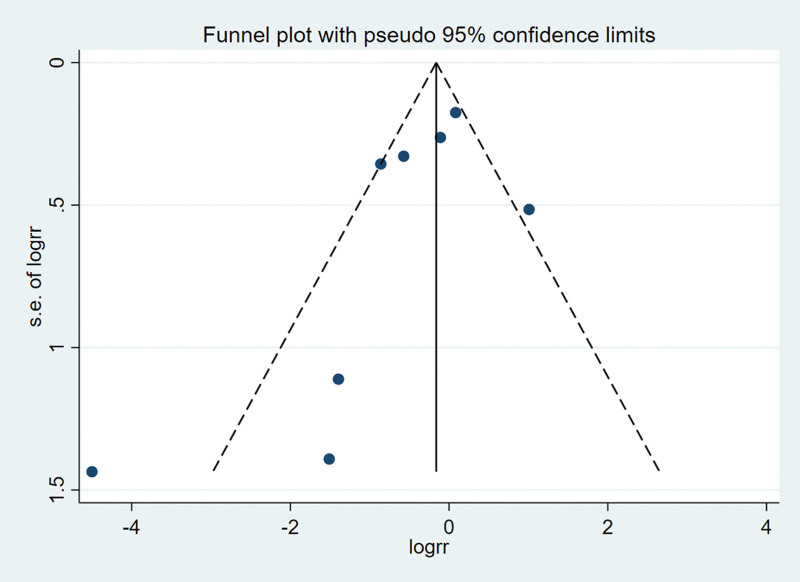
Funnel plot assessing the effect of publication bias on recurrence rate.

**Figure 7.**
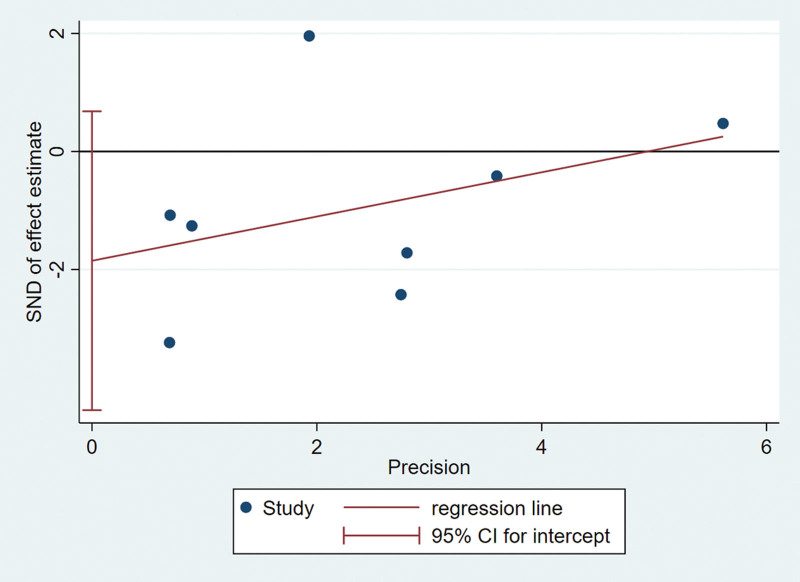
Egger test checks the reliability of funnel plot.

Funnel plots and regression tests were used to test publication bias. The funnel plot is symmetrical with no apparent asymmetry (Fig. 6). In this meta-analysis, the recurrence rate of literatures included in this meta-analysis was not influenced by publication bias as tested by Egger test. (Egger test *P* = .124) (Fig. 7).

## 4. Discussion

In recent years, thyroid cancer has been on the rise worldwide.^[24,25]^ Although the incidence of PTMC has increased dramatically, the mortality rate has remained stable.^[26]^ Therefore, the clinical treatment of PTMC remains controversial. The aim of this study was to compare the clinical outcomes of patients with PTMC as a result of 2 different surgical approaches. A total of 8 studies were included in this meta-analysis, and recurrence rates were lower in both TT groups and LT groups (1.87% and 2.20%, respectively), but Meta-analysis showed that there was no statistically significant difference in the recurrence rate between the 2 surgical procedures.

Based on the findings of our study, we observed a significantly higher surgical risk in the TT group compared to the LT group. The TT group had more hypocalcemia (RR = 4.49) and recurrent laryngeal nerve injury (RR = 36.47). The TT carries the risk of damage to the lateral recurrent laryngeal nerve as well as hypoparathyroidism due to damage to the parathyroid gland. Even experienced surgeons experienced more postoperative complications when performing TT than when performing LT.^[27,28]^ In patients with differentiated thyroid cancer, the primary goal of treatment is to improve survival rates, reduce recurrence. However, reducing vocal and endocrine dysfunction as much as possible is also crucial.^[4]^ In the United States, over the past 25 years, approximately 150,000 females and 50,000 males have undergone TT for PTMC, with thousands of them experiencing parathyroid dysfunction and voice changes.^[29]^ It is therefore too important that the consequences of choosing the best procedure are not influenced by changing treatment habits and worrying about undertreatment.

Our analysis also showed that patients who received TT tended to have slightly better long-term relapse-free survival (HR = 0.57) compared to those who received LT, a finding that may have potential clinical implications in treatment decisions for PTMC. Patients who experience recurrences are susceptible to tissue adhesions, indistinct anatomical layers, heightened intricacy in subsequent surgeries, and an expanded surgical scope may result in a heightened prevalence of postoperative complications.^[30,31]^ RAI can be treated with adjuvant treatment after a TT, considered the most complete procedure. However, in a recent prospective randomized trial, no significant differences in survival rates were found with or without RAI after surgery.^[32]^

Additionally, in terms of postoperative health-related quality of life, LT significantly outperforms TT, possibly due to the higher incidence of complications associated with TT surgery.^[33]^ Furthermore, despite the ability to provide sufficient hormone supplementation, many patients still report symptoms that adversely impact their quality of life, including fatigue, weight gain, and emotional distress.^[34,35]^ The underlying mechanisms of these symptoms remain unclear.

Both the overall incidence comparison and subgroup analysis showed statistical heterogeneity in our meta-analysis. Therefore, we use the random-effects model. Additionally, we performed sensitivity analysis by sequentially removing each individual study in order to test the stability of our results. Egger test was used to verify funnel plot, and no significant change appeared after comprehensive estimation, which strengthened our confidence in the results of meta-analysis.

However, our study also has its shortcomings. First of all, the study did not contain a randomized controlled trial, and all the studies included were cohort studies, which may affect the outcome. In addition, this meta-analysis has the following limitations: Several thyroid LT studies were not matched to controls, so were excluded from this meta-analysis; a few reports were lacking standard deviation data for measurements, which we did not include in our meta-analysis. Because of this part, the number of studies included decreased. As a result, accuracy of the data could be impacted and final results could be biased.

In conclusion, TT was associated with a lower incidence of total complications, recurrent laryngeal nerve injury, and hypocalcemia in the TT compared with LT. In addition, we found a slight improvement in the long-term rate of recurrence-free survival in patients underwent TT compared with those who underwent LT. A finding that has potential clinical implications for future treatment and management decisions for PTMC. To learn more about this, we need to conduct randomized controlled studies on a large sample of patients and multiple medical centers with long-term follow-ups, and with a large sample of medical centers.

## Author contributions

**Conceptualization:** Hao Zhang.

**Data curation:** Jinzhe Bi.

**Formal analysis:** Hao Zhang.

**Funding acquisition:** Jinzhe Bi.

**Investigation:** Jinzhe Bi.

**Methodology:** Hao Zhang.

**Project administration:** Jinzhe Bi.

**Resources:** Jinzhe Bi.

**Software:** Jinzhe Bi.

**Supervision:** Jinzhe Bi.

**Validation:** Jinzhe Bi.

**Visualization:** Jinzhe Bi.

**Writing – original draft:** Jinzhe Bi.

**Writing – review & editing:** Hao Zhang.

## References

[1] McGuire S. World cancer report 2014 Geneva, Switzerland: World Health Organization, International agency for research on cancer, WHO Press, 2015. Adv Nutr. 2016;7:418–9. 26980827 10.3945/an.116.012211 PMC4785485

[2] Davies L Welch HG. Increasing incidence of thyroid cancer in the United States, 1973–2002. JAMA. 2006;295:2164–7. 16684987 10.1001/jama.295.18.2164

[3] Sobin LH. Histological typing of thyroid tumours. Histopathology. 1990;16:513. 10.1111/j.1365-2559.1990.tb01559.x 2361664

[4] Haugen BR Alexander EK Bible KC. 2015 American thyroid association management guidelines for adult patients with thyroid nodules and differentiated thyroid cancer: the American thyroid association guidelines task force on thyroid nodules and differentiated thyroid cancer. Thyroid. 2016;26:1–133. 26462967 10.1089/thy.2015.0020 PMC4739132

[5] Gschwandtner E Klatte T Swietek N. Increase of papillary thyroid microcarcinoma and a plea for restrictive treatment: a retrospective study of 1,391 prospective documented patients. Surgery. 2016;159:503–11. 26189948 10.1016/j.surg.2015.06.015

[6] Creach KM Siegel BA Nussenbaum B. Radioactive iodine therapy decreases recurrence in thyroid papillary microcarcinoma. ISRN Endocrinol. 2012;2012:816386. 22462017 10.5402/2012/816386 PMC3313572

[7] Chow SM Law SC Chan JK. Papillary microcarcinoma of the thyroid-prognostic significance of lymph node metastasis and multifocality. Cancer. 2003;98:31–40. 12833452 10.1002/cncr.11442

[8] Page C Biet A Boute P. “Aggressive papillary” thyroid microcarcinoma. Eur Arch Otorhinolaryngol. 2009;266:1959–63. 19294400 10.1007/s00405-009-0952-5

[9] Cappelli C Castellano M Braga M. Aggressiveness and outcome of papillary thyroid carcinoma (PTC) versus microcarcinoma (PMC): a mono-institutional experience. J Surg Oncol. 2007;95:555–60. 17226813 10.1002/jso.20746

[10] Wang TS Goffredo P Sosa JA. Papillary thyroid microcarcinoma: an over-treated malignancy? World J Surg. 2014;38:2297–303. 24791670 10.1007/s00268-014-2602-3

[11] Rosko AJ Gay BL Reyes-Gastelum D. Surgeons’ attitudes on total thyroidectomy vs lobectomy for management of papillary thyroid microcarcinoma. JAMA Otolaryngol Head Neck Surg. 2021;147:667–9. 33885723 10.1001/jamaoto.2021.0525 PMC8063135

[12] Welch HG Doherty GM. Saving thyroids – overtreatment of small papillary cancers. N Engl J Med. 2018;379:310–2. 30044933 10.1056/NEJMp1804426

[13] Stang A. Critical evaluation of the Newcastle-Ottawa scale for the assessment of the quality of nonrandomized studies in meta-analyses. Eur J Epidemiol. 2010;25:603–5. 20652370 10.1007/s10654-010-9491-z

[14] Parmar MK Torri V Stewart L. Extracting summary statistics to perform meta-analyses of the published literature for survival endpoints. Stat Med. 1998;17:2815–34. Erratum in: Stat Med. 2004 Jun 15;23(11):1817. 9921604 10.1002/(sici)1097-0258(19981230)17:24<2815::aid-sim110>3.0.co;2-8

[15] Duval S Tweedie R. Trim and fill: a simple funnel-plot-based method of testing and adjusting for publication bias in meta-analysis. Biometrics. 2000;56:455–63. 10877304 10.1111/j.0006-341x.2000.00455.x

[16] Hay ID Hutchinson ME Gonzalez-Losada T. Papillary thyroid microcarcinoma: a study of 900 cases observed in a 60-year period. Surgery. 2008;144:980–7; discussion 987. 19041007 10.1016/j.surg.2008.08.035

[17] Lee J Park JH Lee CR. Long-term outcomes of total thyroidectomy versus thyroid lobectomy for papillary thyroid microcarcinoma: comparative analysis after propensity score matching. Thyroid. 2013;23:1408–15. 23509895 10.1089/thy.2012.0463

[18] Donatini G Castagnet M Desurmont T. Partial thyroidectomy for papillary thyroid microcarcinoma: is completion total thyroidectomy indicated? World J Surg. 2016;40:510–5. 26546190 10.1007/s00268-015-3327-7

[19] Dobrinja C Pastoricchio M Troian M. Partial thyroidectomy for papillary thyroid microcarcinoma: is completion total thyroidectomy indicated? Int J Surg. 2017;41(Suppl 1):S34–9. 28506411 10.1016/j.ijsu.2017.02.012

[20] Kim SK Park I Woo JW. Total thyroidectomy versus lobectomy in conventional papillary thyroid microcarcinoma: analysis of 8,676 patients at a single institution. Surgery. 2017;161:485–92. 27593085 10.1016/j.surg.2016.07.037

[21] Kwon H Jeon MJ Kim WG. A comparison of lobectomy and total thyroidectomy in patients with papillary thyroid microcarcinoma: a retrospective individual risk factor-matched cohort study. Eur J Endocrinol. 2017;176:371–8. 28089996 10.1530/EJE-16-0845

[22] Jeon YW Gwak HG Lim ST. Long-term prognosis of unilateral and multifocal papillary thyroid microcarcinoma after unilateral lobectomy versus total thyroidectomy. Ann Surg Oncol. 2019;26:2952–8. 31264119 10.1245/s10434-019-07482-w

[23] Xu Y Xu L Wang J. Clinical predictors of lymph node metastasis and survival rate in papillary thyroid microcarcinoma: analysis of 3607 patients at a single institution. J Surg Res. 2018;221:128–34. 29229118 10.1016/j.jss.2017.08.007

[24] Leenhardt L Grosclaude P. Épidémiologie des cancers thyroïdiens dans le monde [Epidemiology of thyroid carcinoma over the world]. Ann Endocrinol (Paris). 2011;72:136–48. French. 21513910 10.1016/j.ando.2011.03.025

[25] Xiang J Wu Y Li DS. New clinical features of thyroid cancer in eastern China. J Visc Surg. 2010;147:e53–6. 10.1016/j.jviscsurg.2010.02.007 20587378

[26] Lu ZZ Zhang Y Wei SF. Outcome of papillary thyroid microcarcinoma: study of 1,990 cases. Mol Clin Oncol. 2015;3:672–6. 26137285 10.3892/mco.2015.495 PMC4471526

[27] Loyo M Tufano RP Gourin CG. National trends in thyroid surgery and the effect of volume on short-term outcomes. Laryngoscope. 2013;123:2056–63. 23737403 10.1002/lary.23923

[28] Hauch A Al-Qurayshi Z Randolph G. Total thyroidectomy is associated with increased risk of complications for low- and high-volume surgeons. Ann Surg Oncol. 2014;21:3844–52. 24943236 10.1245/s10434-014-3846-8

[29] National Cancer Institute. Surveillance, Epidemiology, and End Results Program (SEER) Database. Available at: www.seer.cancer.gov. [access date March 20, 2023].

[30] Sakorafas GH Giotakis J Stafyla V. Papillary thyroid microcarcinoma: a surgical perspective. Cancer Treat Rev. 2005;31:423–38. 16005155 10.1016/j.ctrv.2005.04.009

[31] Lin JD Chen ST Chao TC. Diagnosis and therapeutic strategy for papillary thyroid microcarcinoma. Arch Surg. 2005;140:940–5. 16230542 10.1001/archsurg.140.10.940

[32] Leboulleux S Bournaud C Chougnet CN. Thyroidectomy without radioiodine in patients with low-risk thyroid cancer. N Engl J Med. 2022;386:923–32. 35263518 10.1056/NEJMoa2111953

[33] Nickel B Tan T Cvejic E. Health-related quality of life after diagnosis and treatment of differentiated thyroid cancer and association with type of surgical treatment. JAMA Otolaryngol Head Neck Surg. 2019;145:231–8. 30653212 10.1001/jamaoto.2018.3870 PMC6439749

[34] Australian Bureau of Statistics Australian Health Survey: First Results 2011–2012. Available at: http://www.abs.gov.au/ [access date June 11, 2018].

[35] Husson O Nieuwlaat WA Oranje WA. Fatigue among short- and long-term thyroid cancer survivors: results from the population-based PROFILES registry. Thyroid. 2013;23:1247–55. 23578315 10.1089/thy.2013.0015 PMC3783928

